# Fibered luminescent concentrator: A bridge between flashlamp devices and laser technologies for skin therapy

**DOI:** 10.1371/journal.pone.0311425

**Published:** 2024-12-18

**Authors:** Catherine Le Blanc, Jean-Luc Perrot, François Balembois

**Affiliations:** 1 Institut d’Optique Graduate School, CNRS, Laboratoire Charles Fabry, Université Paris-Saclay, Palaiseau, France; 2 Department of Dermatology, University Hospital of Saint Etienne, Saint-Etienne, France; 3 Laboratoire Hubert Curien, CNRS, Université Jean Monet, Saint-Etienne, France; 4 Laboratoire de Tribologie et Dynamique des Systèmes, CNRS, Saint-Etienne, France; Universiti Teknologi Malaysia - Main Campus Skudai: Universiti Teknologi Malaysia, MALAYSIA

## Abstract

**Background and objectives:**

Laser skin therapy and intense pulsed light (IPL) therapy are both light-based treatments used for various skin concerns. They have been used since decades and each system have their own specificity, advantages, and drawbacks. However specific treatment is still not accessible with standard techniques due to difficulties having a source with both laser and IPL advantages. We describe a new concept, the fibered luminescent concentrator—FLC, based on luminescent concentrators capable of concentrating spectrally and spatially an IPL source, resulting in a multi-color fibered device.

**Study design/materials and methods:**

The FLC utilizes luminescent materials arranged in parallelepiped shapes polished on all faces. The IPL broadband spectrum is absorbed by the luminescent molecules and is re-emitted to a red shifted wavelength. The emitted spectral bandwidth ranges from green to dark red, depending on the type of luminescent concentrator. This light is then spatially concentrated by total internal reflections in the parallelepiped and guided through a fiber to the final operator.

**Results:**

We have developed three different solid luminescent concentrators based on a transparent polymer sheet (PMMA) doped with luminescent organic dye molecules for yellow and red emission, and an alexandrite crystal for emission in the dark red spectrum. We demonstrate that our new non-laser FLC device can concentrate spectrally and spatially the light with no temporal deformation and offers real opportunities for treatments where the IPL is less well-adapted.

**Conclusion:**

The FLC is an additional tool for existing conventional systems such as laser or IPL sources. It is easily adaptable to any IPL source and is a very good complement, especially for wavelengths where the laser cannot easily produce light, such as the yellow band.

## Introduction

Lasers and intense pulsed light (IPL) technologies are the two main techniques using visible light in dermatology [1]. The photochemical effect on the skin occurs through the interaction of light—a laser or an IPL—with specific excitation bands of chromophores, molecules or photosensitizers of precise cellular structures [2]. Understanding the impact of visible light involves examining how various tissue and blood components absorb light across the spectrum from 200 nm to 800 nm. As cited in reference [3], dermatologic laser therapy has traditionally focused on three fundamental chromophores: hemoglobin, melanin, and water. Hemoglobin and oxyhemoglobin exhibit distinct absorption peaks at 400 nm (in the blue spectrum) and 580 nm (in the yellow spectrum) respectively. Melanin, on the other hand, absorbs light throughout the entire visible spectral range, spanning from 400 nm to 800 nm, while water does not exhibit significant absorption within the visible range.

### Laser versus non-coherent IPL sources

Lasers and non-coherent IPL sources are based on the principle of selective photo-thermolysis and can be used for the treatment of many vascular skin lesions. While these two light-based technologies exhibit some similarities, they also possess distinct characteristics. E. Victor Ross’s review paper entitled "Laser versus Intense Pulsed Light" highlights their competitiveness within the field of dermatology [1].

On one hand, lasers offer exceptional brightness attributed to their coherence and the ability to focus light tightly. They can be efficiently coupled into optical fibers, enabling remote light delivery to the treatment area. This results in a more precise and targeted treatment range. Lasers can be tuned to specific wavelengths, allowing them to selectively target chromophores, such as hemoglobin and oxyhemoglobin in the skin. This feature makes lasers well-suited for procedures like skin resurfacing and acne treatment [4–6]. However, lasers come with limited versatility as they operate at fixed wavelengths, including 650–690 nm, 800 nm, 1470 nm for laser diodes, 1064 nm and 532 nm for Nd:YAG lasers, and 755 nm for alexandrite or 694 nm for ruby lasers. Notably, there is a lack of lasers emitting in the yellow spectrum, except for dye lasers, which can be challenging to handle. Another consideration is that laser light is a coherent light often collimated, making it less safe for the eyes compared to IPLs.

**Fig 1:**
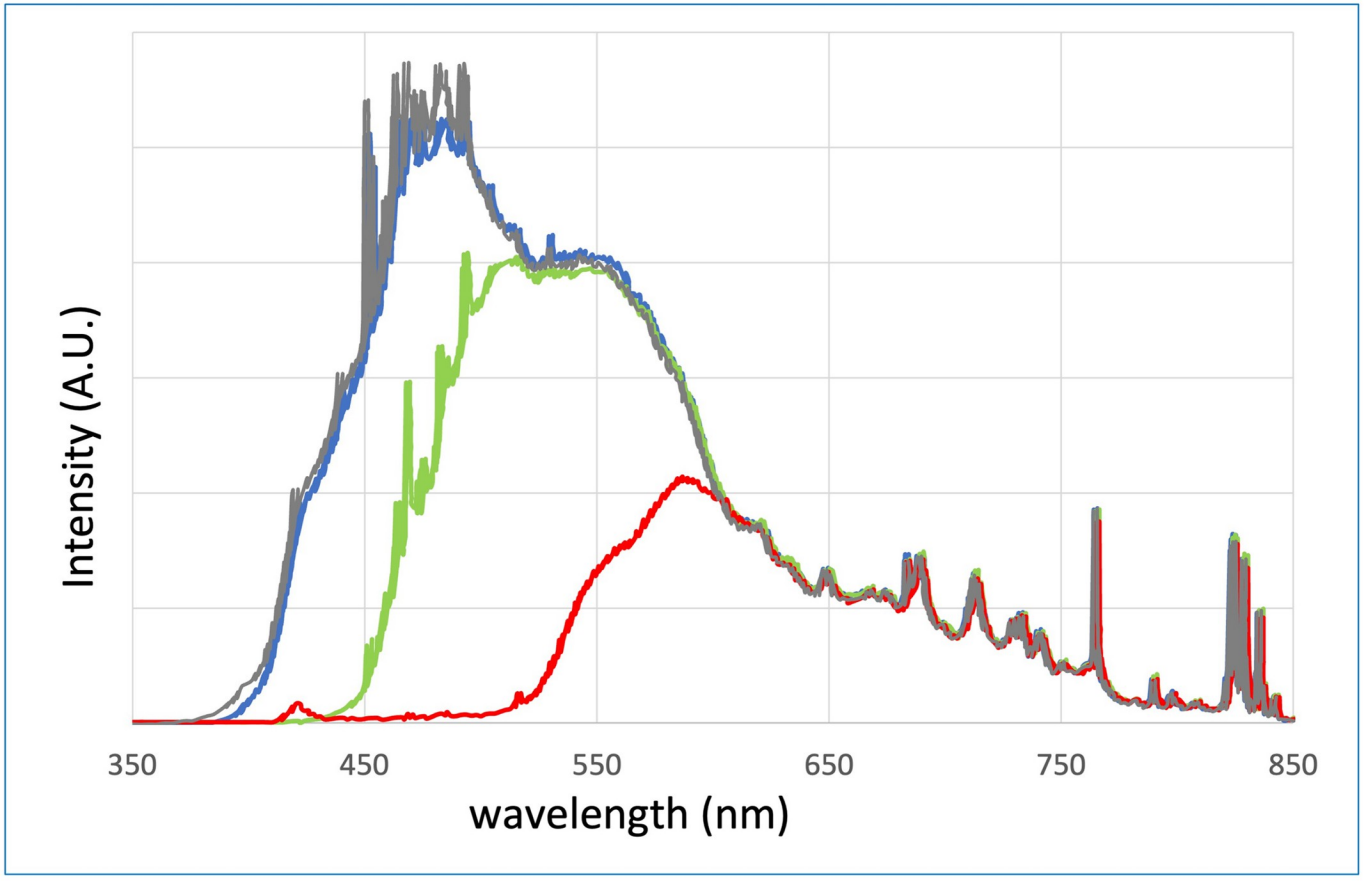
Spectra MED-230. Spectra measured using our IPL system (MED-230 from Beijing KES Biology Technology Co.) with various filters positioned after the flashlamp in the handpiece. The grey curve is the flashlamp spectrum without any filter. The blue (420 nm), green (530 nm), and red (630 nm) curves are the spectra with the high-wavelength bandpass absorption filters.

IPL sources tend to have wider margins when it comes to light safety limits. The inherent beam divergence of IPLs makes them less likely to pose a direct eye hazard as compared to lasers. Nonetheless, there have been instances of eye injuries in patients following IPL treatments, and it is advisable for both the operator and the patient to use eye protection, as noted in reference [1]. The substantial divergence and large treatment surface area (usually 5–10 cm^2^) of IPL systems make them well-suited for addressing extensive areas of the skin, particularly in the context of hair removal [7]. In many dermatological applications that require millisecond (ms) or longer pulse deliveries over significant skin areas, IPLs prove to be not only adequate but even preferable to lasers. Assessing the skin’s response to IPL is a more intricate task compared to laser radiation. IPL therapy utilizes a broad spectrum of visible light, allowing it to address a wider array of skin concerns through appropriate spectral filtering [8, 9]. Most IPL systems employ dichroic filters, which are essentially long-pass transmission filters with broad and partially non-selective spectra, resulting in skin heating. In addition, the light that is not transmitted by the filters is either reflected in the IPL head or absorbed in the filters. In all cases it adds additional heat in the flashlamp head. As an example, Fig 1 displays the transmission spectra for each filter within our IPL system (MED-230 from Beijing KES Biology Technology Co.). It’s important to note that these curves are presented without any treatment (as measured). Ultimately, these filters introduce some losses in the short-wavelength domain.

An additional challenge arises as the IPL spectrum depends on the age of the flashlamp and on the driving conditions (current and voltage). Consequently, even with spectral filters, IPL spectrum variation can affect treatment parameters (namely fluence in J/cm^2^ and temporal pulse shape). When working with IPL devices, the operator is faced with the challenge of managing the lamps, lamp cooling system, and high voltage wires, all housed within a bulky handpiece connected to a power supply via a thick and often rigid "umbilical cord." The typically large handpieces and wide spot sizes of IPL systems can also hinder maneuverability, especially when treating uneven or irregular skin surfaces. The Cutera^®^ manufacturer has managed to address these limitations effectively. They have developed a "small" IPL source with a 6 mm spot size (Cutera AcuTip Handpiece) for treating discrete brown and red dyschromias as described in [1]. This innovation involves the use of a long cylindrical sapphire waveguide and high-performance reflectors, yet it still results in a heavy handpiece.

### Other techniques of non-coherent sources

We should also mention two specific techniques that originate from non-coherent light sources. The first technique, known as TRASER (Total Reflection Amplification of Spontaneous Emission of Radiation), was pioneered by C. Zachary and M. Gustavsson in 2012 [10]. TRASER relies on the concept of spontaneous emission from dyes (or solids) that are triggered by flashlamp pumping and then confined through total internal reflection within a fiber. This type of concentrator with fiber geometry is very well detailed in reference [11]. In TRASER, there exists an internally reflecting structure that houses a fluorescent substance referred to as the "dye cell." Flashlamps are strategically positioned around this dye cell. The internally reflected photons propagate axially along the length of the dye cell or crystal towards the output surface. At the proximal end (opposite to the output surface) of the cell, a mirror redirects the emitted light forward, and this light is subsequently passively extracted. A waveguide is employed to precisely target the treatment area. This TRASER system has demonstrated good results, as documented in several publications [12–14].

**Fig 2:**
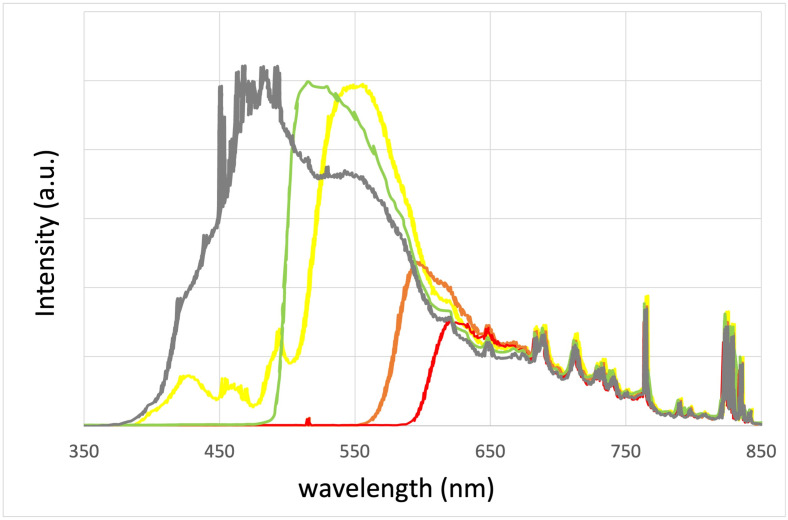
Spectra with fluorescent filters. Output spectra measured using our IPL system (MED-230 from Beijing KES Biology Technology Co.) with various fluorescent filters: green (520 nm), yellow (550 nm), orange (590 nm) and red (620 nm) filters (PMMAs from PERSPEX^®^), positioned after the flashlamp in the handpiece.

The second technique, named Advanced Fluorescent Technology (AFT) [15] or Fluorescent Pulsed Light (FPL) [16], is founded on a standard IPL system enhanced with a fluorescent filter. This filter absorbs IPL flashlamp light and re-emits it selectively across a spectrum that depends on the characteristics of the filter. Therefore, the light after the filter has two components: the light of the IPL that is transmitted by the filter and the spontaneous emission of the filter. As an example, the 650–950 nm AFT handpiece has a filter which converts the IPL light into a clinically optimal spectrum by selectively emitting in the red spectrum (as referenced in [15]). It has been effectively employed in the treatment of refractory melasma in Asian patients, as described in reference [16]. Fig 2 illustrates the expected spectrum when using fluorescent filters. The spectra closely resemble those measured with standard filters, as depicted in Fig 1, except for the "bump" on each curve corresponding to the fluorescence of the filter.

Before introducing our novel source, the Fibered Luminescent Concentrator (FLC), which combines the precision of lasers with the spectral flexibility of IPL, we outline the fundamental principle of a luminescent concentrator.

### Luminescent concentrator

A luminescent concentrator is a broadband source that provide power and brightness. It has been recognized and utilized for over half a century, primarily in the context of luminescent solar concentrators for the generation of photovoltaic energy [17]. It typically consists of a rectangular slab embedded with fluorescent luminophores, with its larger surface area exposed to sunlight or any other non-coherent light source. Recent research [18, 19] has demonstrated remarkable performance in luminescent concentrators pumped by LEDs, where the collected light is directed along one of the shorter faces of the parallelepiped concentrator.

The process involves the absorption of pump light by the fluorescent luminophore, which then emits lower-frequency photons within the bulk material. These emitted photons are guided through the material by total internal reflection until they reach the smaller surfaces. This results in an augmented output irradiance, directly correlated with the ratio between the large surface area and the area of the edge face.

#### 3D-luminescent concentrator

**Fig 3:**
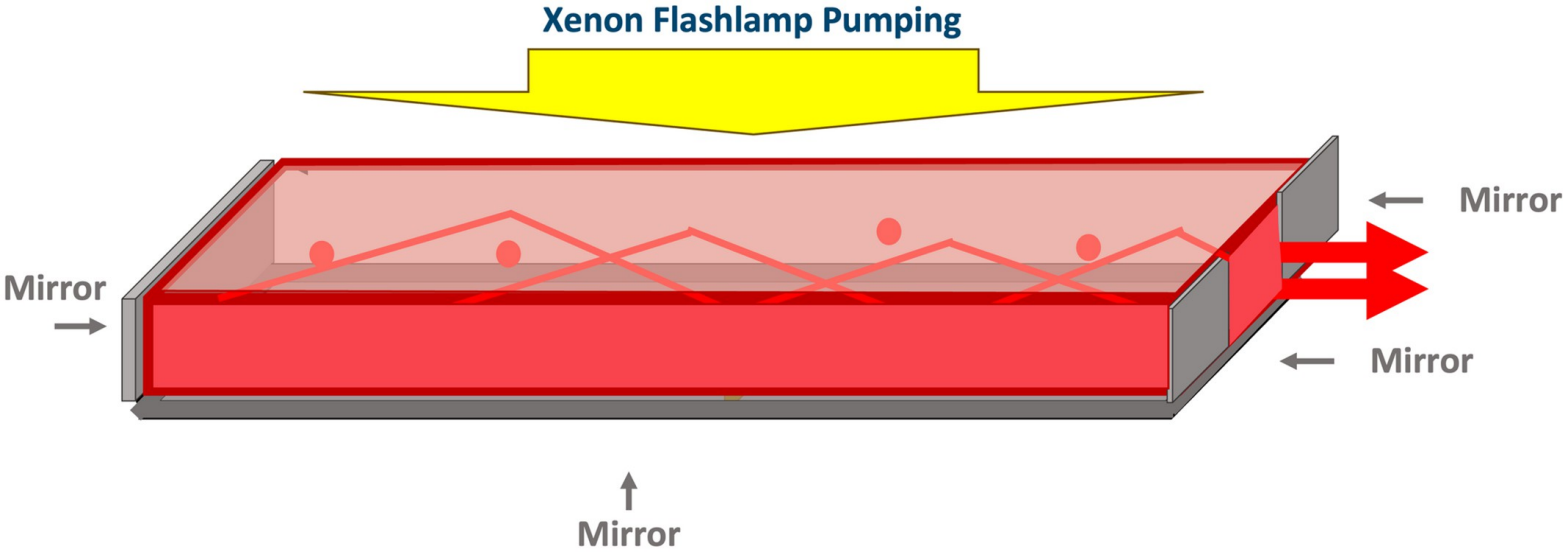
3D luminescent concentrator concept. The 3D configuration involves adding mirrors to the output face and to the opposite face to enhance brightness. In this setup, one additional mirror is placed under the luminescent concentrator to recycle the pump.

To enhance extraction efficiency, a 3D luminescent concentrator, as described by Pichon et al. [20] and illustrated in Fig 3, has been developed. It relies on light recycling within the structure, achieved by adding mirrors to a portion of the output face and to the opposite face. All photons hitting the output surface at the mirror locations are reflected and redirected back into the concentrator until they finally reach the output surface, despite some losses. The 3D luminescent concentrator has demonstrated a remarkable increase in brightness by one order of magnitude.

#### Fiber-coupled luminescent concentrator

Previously, some work using polymer a fiber-coupled luminescent concentrator has been demonstrated [21]. Luminescent solar concentrators with cylindrical geometry are modeled [22], and the problem of re-absorption is well studied. Our approach is different, as we separate the spectral and spatial concentration (using a luminescent concentrator) and the light guiding (using a CPC and a transparent liquid light guide). This setup allows us to guide a significant amount of energy with no limitation in terms of fiber length. In our case, the re-absorption effect is taken into account and is not an issue, as shown in the results and discussion. More recently, a luminescent concentrator has also been coupled into optical fibers for low-energy applications such as medical diagnostics, agriculture, or telecommunications [23]. However, our setup is optimized for delivering high energy, comparable to IPL or laser skin therapy.

#### Fibered luminescent concentrator

For a light source utilized in dermatology, efficient beam delivery to the treatment area is crucial. Laser sources are often fiber coupled for this purpose, offering a user-friendly light tool.

However, due to the large size of a flashlamp, fiber coupling of an IPL is inefficient, requiring the flashlamp to be positioned close to the skin. This results in a bulky handpiece, a drawback that could be mitigated by our luminescent concentrator. For this purpose, we employed to couple the output light of a luminescent concentrator into a light guide, named it as Fibered Luminescent Concentrator. We describe the setup in the next paragraph.

## Materials and methods

### Luminescent concentrator material

In our study, we employ two distinct types of materials. The first category uses two colors, one yellow (reference 1C50 GT), and one red (reference 3CO2 GT), all from Plexiglas^®^ GS and made from fluorescent cast acrylic (Polymethylmethacrylate-PMMA). These PMMA-based concentrators consist of fluorophores embedded within the PMMA matrix. The PMMA concentrators are 50 mm long by 15 mm wide, adapted to the size of our IPL handpiece and are 3 mm thick. Note that we do not have access to the exact concentration of fluorophores. They are laser-cut and polished on all sides in our laboratory.

The second type of concentrator we use is a chromium-doped alexandrite crystal, known for its laser properties. To the best of our knowledge, this is the first time it is being used as a luminescent concentrator, i.e., for spontaneous emission rather than stimulated emission. This alexandrite luminescent concentrator consists of two crystals polished on all sides, bonded together to match the dimensions of the IPL handpiece. While monolithic alexandrite with the appropriate dimensions could have been selected, we opted for smaller alexandrite crystals available in our laboratory (20 mm long x 10 mm wide x 2 mm thick), originally designed to function as a laser medium, to assess the versatility of the concept. The crystals have a chromium doping level of 0.22% and are provided by Northrop Grumman.

### Method for setting up the fibered luminescent concentrator

We present the integrated system, which was used in conjunction with two IPL handpieces.

**Fig 4:**
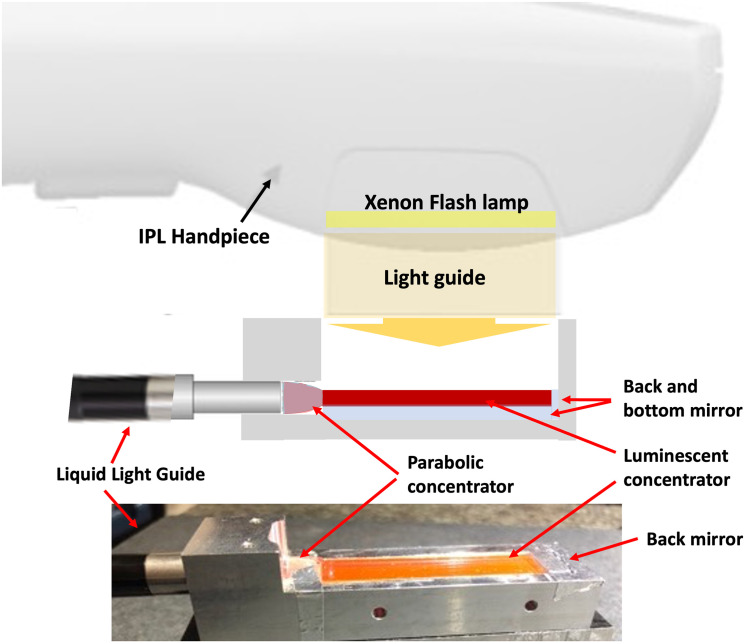
Schematic (and picture) of our fibered luminescent concentrator—FLC. The luminescent concentrator has the same size as the sapphire guide from the handpiece. In our case, for optimizing efficiency, it is 50mm long and 10mm wide. To recycle the unabsorbed light, a mirror is placed at the bottom of the device. A compound parabolic concentrator, glued to the smallest side, facilitates the extraction of concentrated energy and the adaptation of numerical aperture. The 5 mm diameter concentrated light then travels through a liquid light guide to reach the final user.

The experimental setup is shown in Fig 4 Starting with the IPL handpiece as the light source, we position a luminescent concentrator in close contact with the xenon flashlamp light. In the case of our two IPLs, the handpiece incorporates a sapphire crystal to guide the xenon flashlamp light towards the skin. Concentrators dimensions are nearly the same: 10 mm x 50 mm for the MED-230 and 10 mm x 48 mm for the Nordlys^®^ equipped with a PL 400 handpiece. The luminescent concentrators are designed to match the pump area. To optimize efficiency, we have strategically placed mirrors around the concentrators. One mirror is situated above the LC to recycle any unabsorbed light and two additional mirrors are positioned on both sides to confine the light in the three spatial dimensions and to increase the brightness. The recycling of the light using the back and front mirrors increases the efficiency by 60%. Note that due to re-absorption in the medium, we do not double the energy, as mentioned in [20].

The emitted light exhibits significant divergence and is very close to a Lambertian source [19] with a numerical aperture close to 1. Direct coupling in fibers (with typical numerical aperture below 0.6) would results in large losses. To address this challenge, we have implemented a solution involving a collimator known as a molded compound parabolic concentrator (CPC) from Edmund Optics as detailed in [24]. This collimator reduces the numerical aperture at a cost of beam increase (up to 5 mm diameter). At the output, the numerical aperture is 0.6, adapted to a light guide (LLG5 from Thorlabs^®^). As a result of this setup, the beam diameter at the output of the fiber measures 5 mm, ensuring an effective and controlled light output.

**Fig 5:**
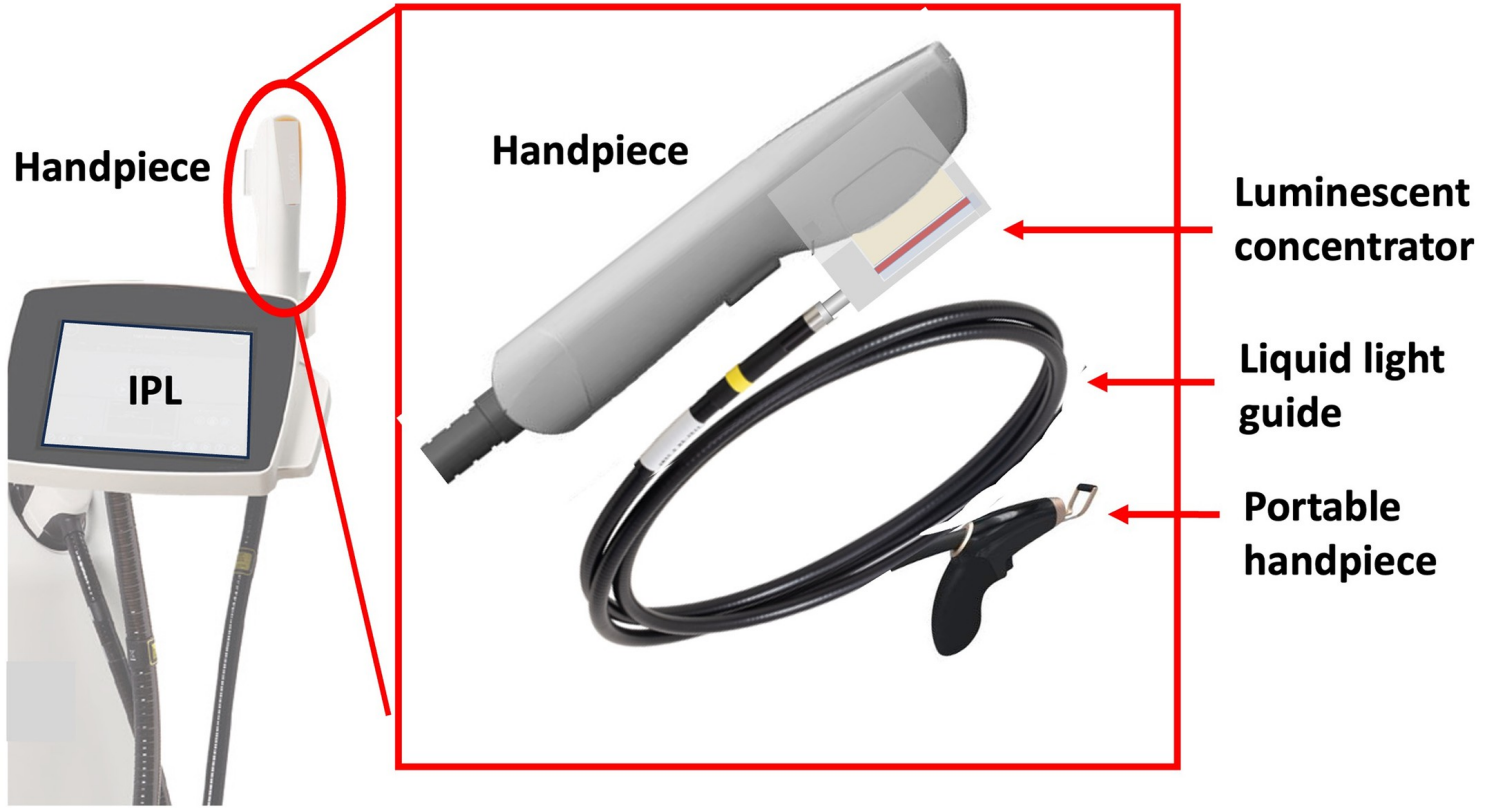
Schematic of the system attached to the IPL.

Fig 5 gives a view of the complete system, including the IPL used as a pump source. It’s worth noting that the most suitable handpieces for this application are those that do not incorporate any type of filter. This is because the flashlamp pump requires the delivery of a full spectrum of light to be effectively absorbed by the concentrators. We secured our FLC to the handpiece. The concentrated light is transmitted through the 1.5 m long light guide. At the end of the light guide, we have attached a small, lightweight handpiece for the end user. The output beam has a diameter of 5 mm with a numerical aperture of 0.6.

## Results and discussion

### Luminescent concentrators characterization and performance

The three concentrators have been selected with the aim of addressing primary applications that are wavelength dependent. The yellow PMMA emits light at a peak wavelength of 580 nm, which coincides with the absorption spectrum of oxyhemoglobin. This characteristic makes it particularly well-suited for applications involving oxyhemoglobin absorption. The red PMMA emits light at wavelengths beyond the absorption peaks of both hemoglobin and oxyhemoglobin. Consequently, it proves to be a suitable choice for applications such as hair removal. Finally, the alexandrite crystal has already demonstrated a wide range of applications in the field of dermatology. Note that this technique is extendable to other laser materials and other PMMAs (green, orange, dark red) for specific applications, provided their absorption spectrum coincides with the emission spectrum of the xenon flash lamp used in IPLs.

**Fig 6:**
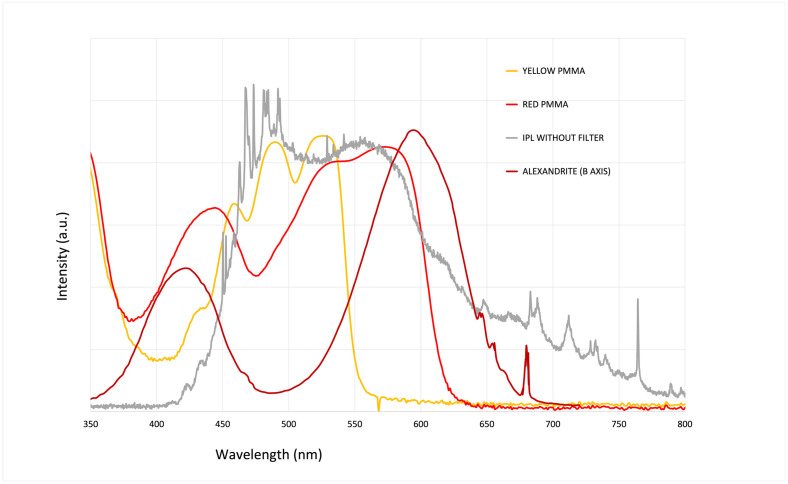
Absorption spectra from our 3 luminescent concentrators compared to IPL emission spectrum. We normalized spectral absorption profiles of the three concentrators used in our setup. The spectral absorption range of yellow PMMA is centered at 500 nm, while red PMMA is centered at 600 nm. The alexandrite crystal, oriented along its B axis, exhibits two absorption peaks, one centered at 400 nm and the other at 600 nm. The grey curve represents the broadband emission spectrum of the IPL xenon flashlamp.

Fig 6 shows the absorption wavelength ranges of each material used in our study, including the red and yellow PMMAs and the alexandrite crystal. The three absorption curves, overlaid with the IPL flash lamp emission spectrum. Overall, the red PMMA covers the broadest emission bandwidth of the IPL.

**Fig 7:**
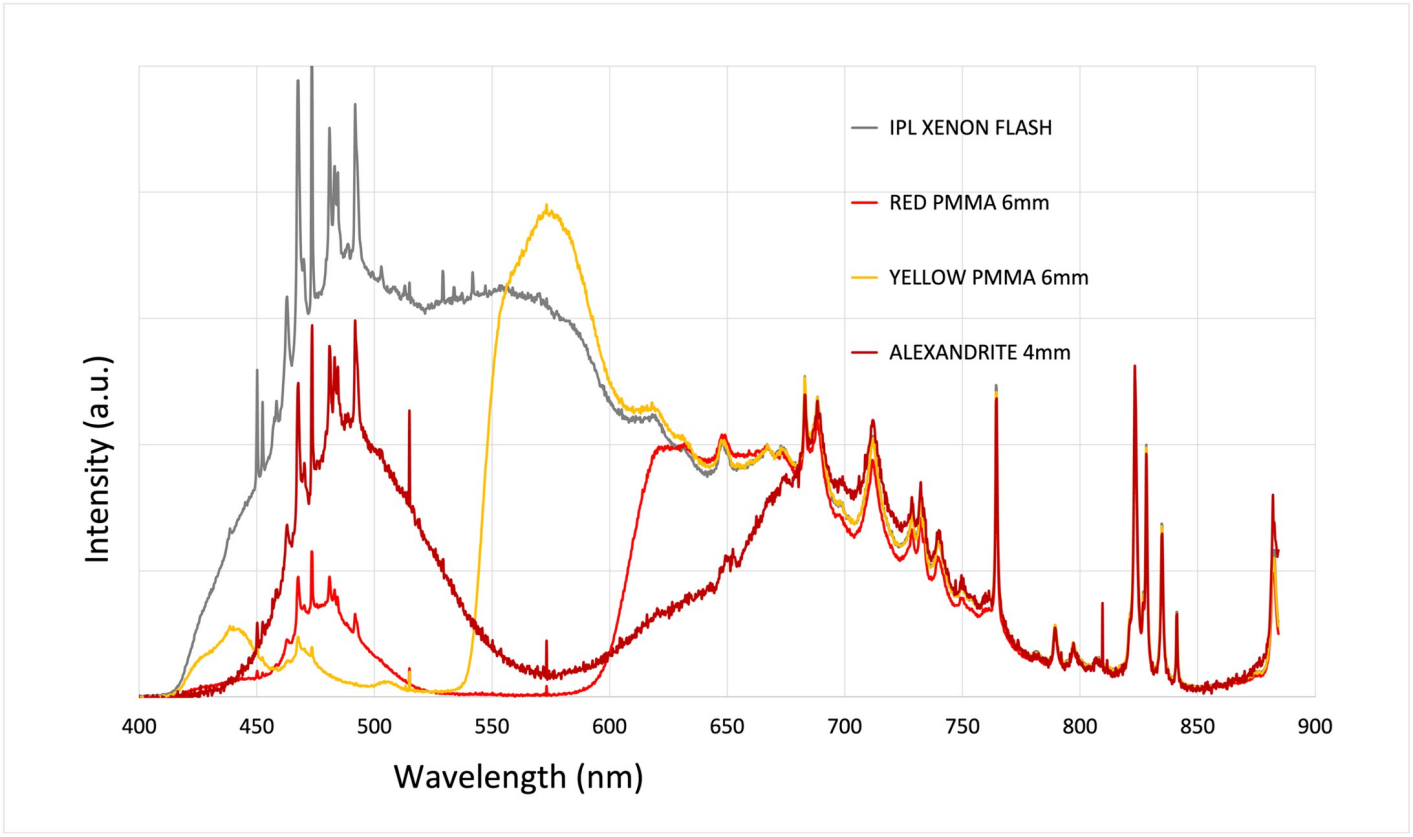
Transmitted spectra from the three luminescent concentrators through the IPL flashlamp compared to the emitted flashlamp spectrum represented in grey. Each concentrator is placed under the IPL handpiece. We doubled the thickness of each concentrator to account for the effect of the recycling mirror. The final thickness of our concentrators is 6 mm for PMMAs and 4 mm for the alexandrite. The reference spectrum is the IPL xenon flash lamp emission in arbitrary unit and all other measurements are done using the same level of IPL emission. The measured spectra after passing through each concentrator correspond to the absorption and re-emission of each material.

To evaluate the spectral efficiency of the flashlamp pumping, we measured the absorption of each concentrator in our system. The absorption for the red PMMA, the yellow PMMA and the alexandrite are respectively 50%, 30% and 30%. As shown in Fig 3, one additional mirror is placed under the luminescent concentrator to recycle the pump. Consequently, the final transmission measurement was also conducted using double the thickness for each concentrator. The resulting absorption for the red PMMA (6mm), the yellow PMMA(6mm) and the alexandrite (4mm) are respectively 60%, 34% and 50% (Fig 7). The measured spectra for each concentrator tend to zero where the absorption occurs. This behavior indicates that nearly maximum absorption is achieved using this recycling technique, proving that our PMMA fluorophore concentration and chromium concentration in alexandrite is optimal. The chromium concentration in the alexandrite is already the highest we could obtain (0.22%).

**Fig 8:**
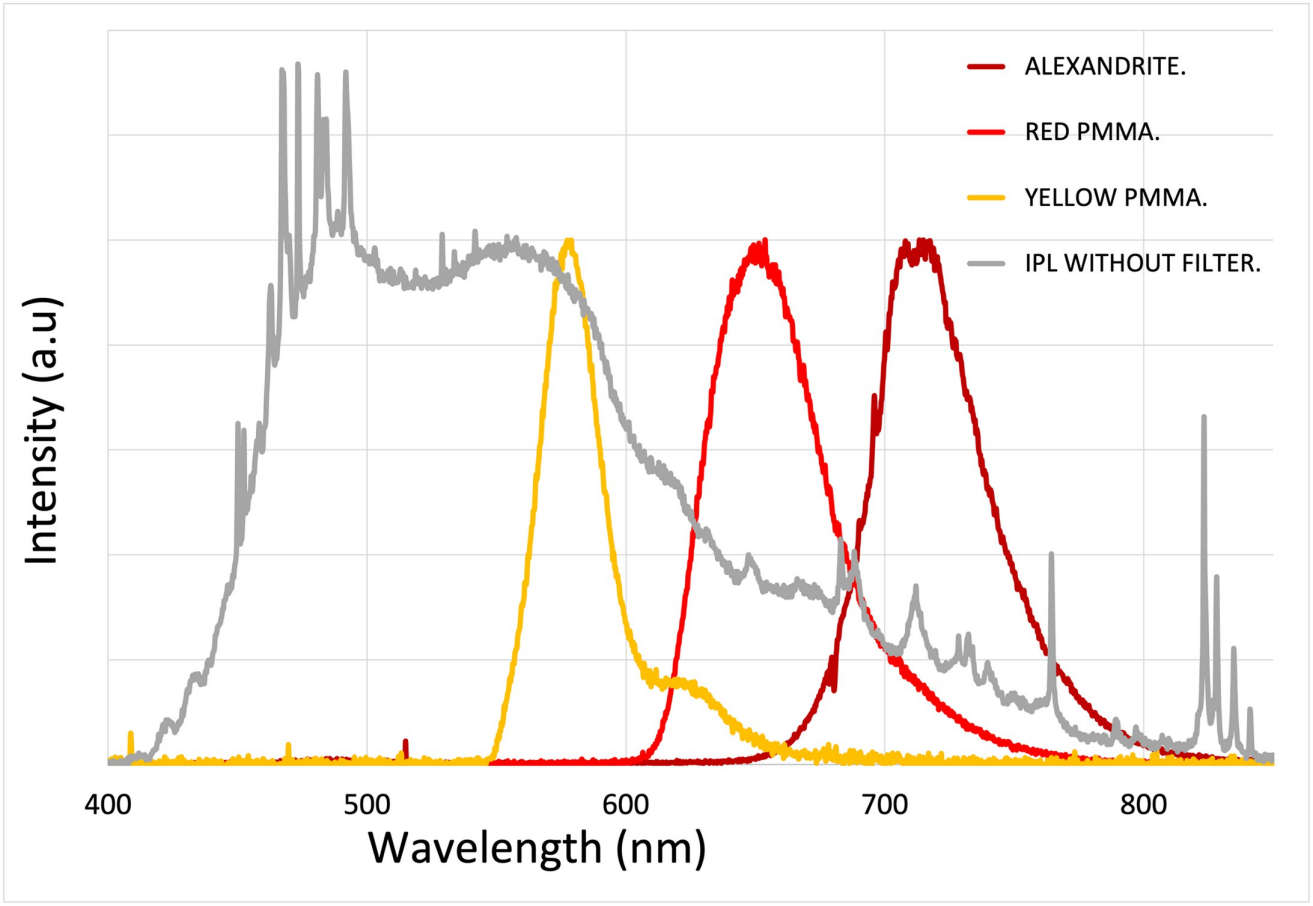
Spectra from IPL and luminescent concentrators. Output Normalized spectral emission profiles of our IPL system (MED-230 from Beijing KES Biology Technology Co.) without any filter (grey curve) compared to the normalized spectral emission of the three luminescent concentrators used in our setup. The yellow PMMA spectrum is centered at 580 nm with a full-width-at-half-maximum (FWHM) bandwidth of 28 nm, the red PMMA at 655 nm with a FWHM bandwidth of 50 nm, and the alexandrite crystal emits at 715 nm with a FWHM bandwidth of 50 nm.

By utilizing a broad-spectrum IPL flash (MED-230 from Beijing KES Biology Technology Co.) as the pump source for all three concentrators, we have successfully achieved spectral shifts and concentrated the light to suit the specific applications described earlier. Fig 8 displays the spectral emission profiles for each concentrator, alongside a typical xenon flashlamp spectrum commonly used in IPLs.

We have tested that the emission spectra of the three luminescent concentrators remain consistent regardless of the pump power level or even when using two different IPL devices: MED-230 from Beijing KES Biology Technology Co. and Nordlys^™^ from Candela^®^. This represents a key advantage as the spectrum produced by luminescent concentrators remains unaffected even when the flashlamp ages or is replaced. This stability is a notable advantage in practical applications.

### FLC performance

**Fig 9:**
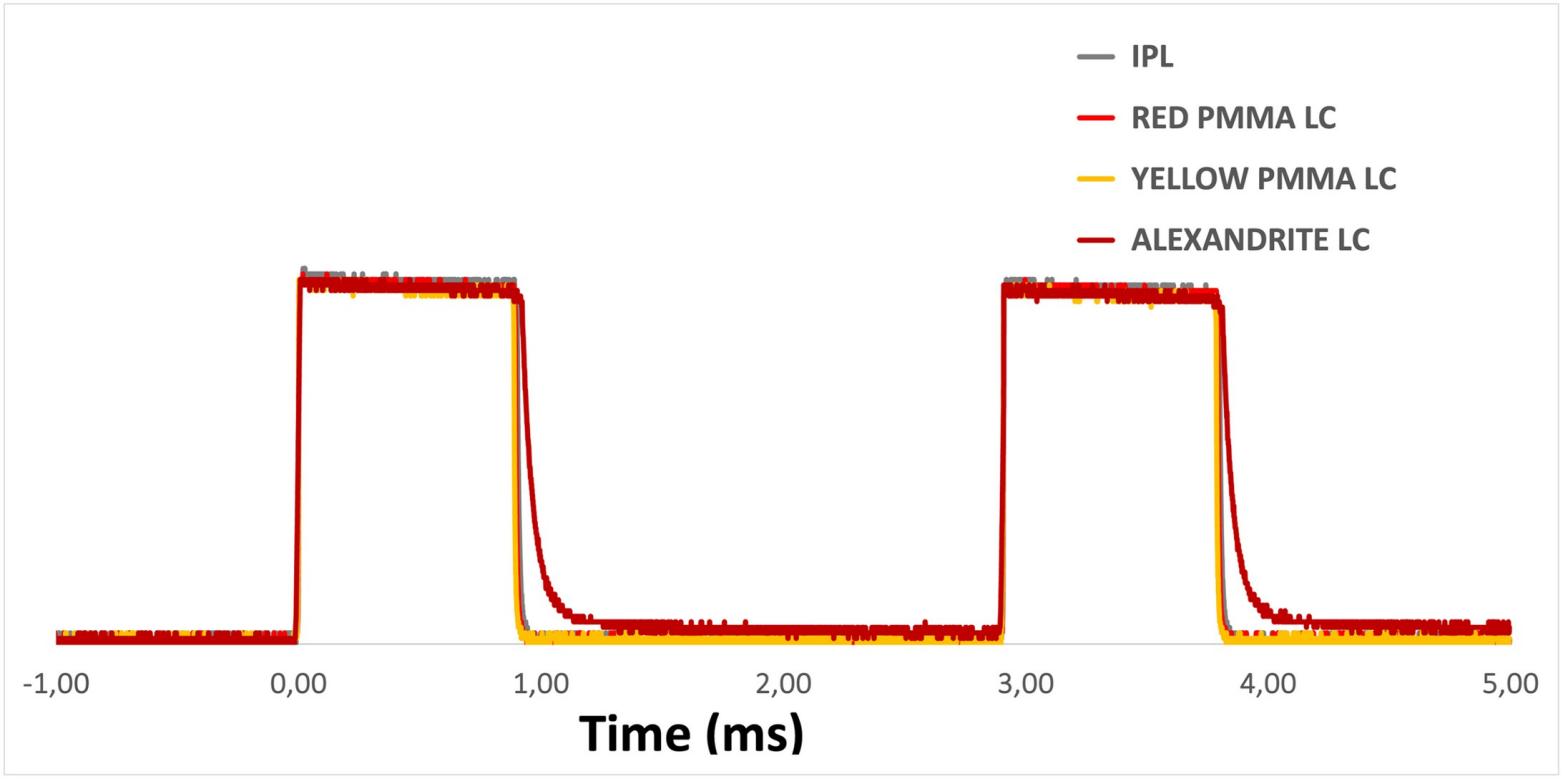
Temporal measurement. Measurement of the temporal characteristics of the emitted light compared to the flashlamp’s temporal profile. We pumped our FLC with a temporal window of 1ms. The experiments were conducted using yellow and red PMMAs and the alexandrite.

Earlier, we demonstrated that our FLCs serve as spectral concentrators. It is essential to verify that this spectral concentration does not alter the temporal characteristics of the emitted light. We conducted numerous experiments on both PMMAs and the alexandrite crystal, and compelling evidence supporting this is presented in Fig 9. During these experiments, we applied various temporal shapes to the light source and used a fast photodiode to measure the temporal profile at both the output of the IPL handpiece and at the exit of our fiber optic. For both PMMAs, our findings indicate that the temporal shape of the light remains unaltered, in accordance with their very short lifetime of few tens of ns. For the alexandrite crystal, we observed a slight increase in the temporal window due to its fluorescence lifetime of 260 μs.

We evaluated the performance of our FLC using the two different IPL systems, first the IPL MED-230 and secondly the Nordlys^®^.

With the IPL MED-230 system, we could adjust the fluence up to 27 J/cm^2^ for a pulse duration between 2 ms and 9 ms. We quantified the energy output using an energy meter (Gentec, model QE95LP-S-MB-QED-DO) for pulse duration up to 2 ms. For measurements involving longer pulses ranging from 3 ms to 9 ms, we substituted the joule meter with a power meter (Gentec, model UP55N-50S-VR-DO). These measurements were performed at a repetition rate of 2 Hz for a duration of 10 seconds, resulting in 20 shots per measurement. The measurements indicate a root mean square fluctuation of 3% related to the flashlamp stability.

**Fig 10:**
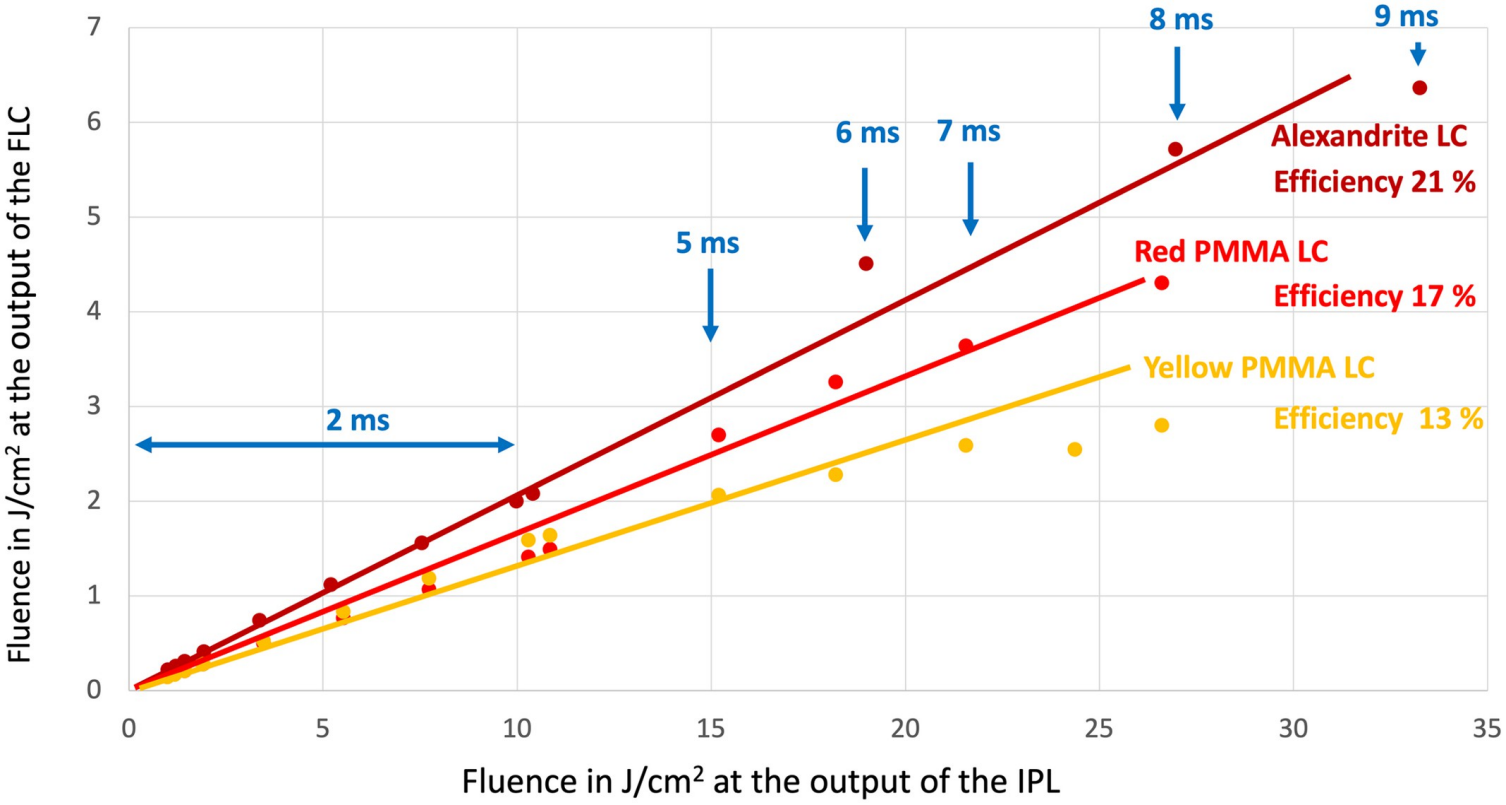
Output fluence of FLCs with MED230. Experimental results were obtained for the three FLCs (alexandrite, red and yellow PMMA concentrators). The IPL MED 230 was used with the 50 x 15 mm^2^ handpiece, without any filter. Up to a pump fluence of 10 J/cm^2^, the fluence is varied by adjusting the flash intensity, the pulse duration remaining at 2 ms. This allows us to use a Joule meter (Gentec). Upon reaching 10 J/cm^2^, the fluence is increased by widening the temporal window up to 9 ms.

For each type of FLC, we successfully achieved fluences exceeding 5 J/cm^2^ in a single shot with a pulse duration of 9 ms. Fig 10 presents the results obtained for the three FLC colors (PMMA yellow, red, and alexandrite) under these experimental conditions.

The efficiency in terms of fluence ranges from 13% to 21%, depending on the material used for the concentrator. The difference of performance between the alexandrite and the PMMA can be related to propagation losses that are higher for PMMA than for a crystal laser such as alexandrite. We also notice that the red PMMA gives better results, probably due to a better fit of the absorption curve with the flashlamp spectrum, compared to the yellow one (see Fig 10). For the highest pump fluence (starting at 25 J/cm^2^), the yellow PMMA tends to lose energy. We have noticed that the heating effect starts to become problematic when operating at this fluence level. The heating effect on the concentrator results in a performance loss. This loss can be reversible up to a certain limit (25 J/cm^2^, 10 shots, 10s). Beyond this value, bubbles form on the surface of the plastics and permanently damage the surface. In this case, simply replacing the plastic is sufficient with almost no cost. Another solution could be to reduce the repetition rate or to widen the temporal window to deliver the same energy over a longer duration.

Concerning the alexandrite crystal, we observed no damage while pumping up to the maximum (25 J/cm^2^, 100 shots).

This moderate efficiency is the "price to pay" for achieving spatial concentration, which enables fiber coupling and offers a handpiece that is much easier to handle than the IPL handpiece.

**Fig 11:**
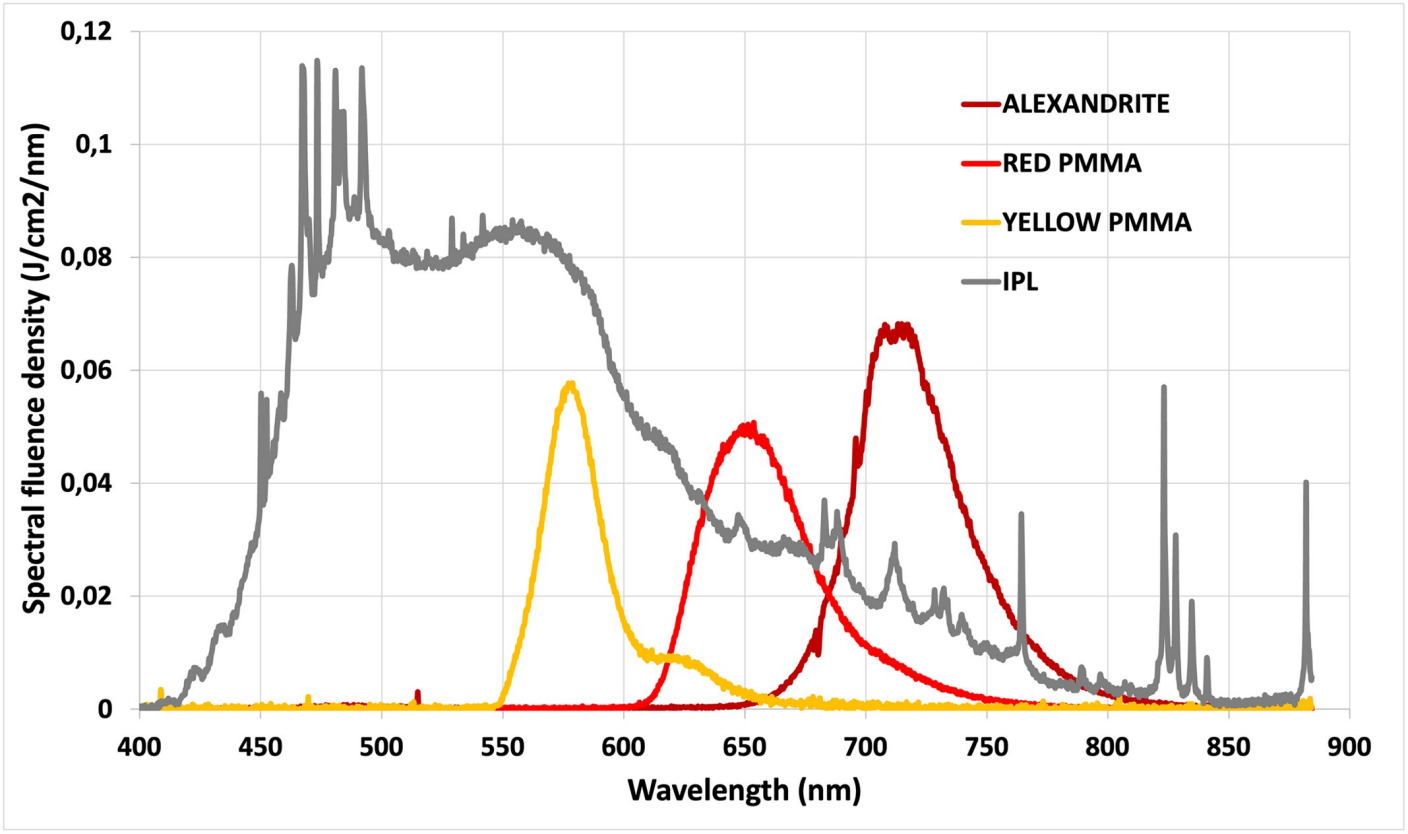
Spectral fluence density of FLCs. Spectral fluence density in (J/cm^2^/nm) measured for the IPL flashlamp and for each fibered luminescent concentrator (red, yellow and alexandrite). This measurement has been carried out for a pump fluence of 18.5 J/cm^2^, using the IPL MED 230 handpiece.

To complete the comparison of FLC with IPL, one must consider the emitted spectrum. In Fig 11, we have plotted the different spectral emissions of our three FLCs and compared them to the spectral density of fluence in J/cm^2^/nm of the xenon flashlamp from the IPL. The measurement was done for a pump fluence of 18.5 J/cm^2^, and according to Fig 10, the output fluence measured for the yellow, red, and alexandrite FLCs are respectively 2.3 J/cm^2^, 3.3 J/cm^2^, and 4.5 J/cm^2^.

As mentioned previously, the efficiency is below 25% for all FLCs, but this does not take into account the effect of spectral concentration. Indeed, Fig 11 illustrates that at the peak wavelength of each FLC, the spectral fluence density is higher for the red PMMA (170%) and for the alexandrite (420%) than for the IPL. The yellow PMMA achieves a peak efficiency of 75% compared to IPL. Compared to passive filters, a luminescent concentrator can surpass a flashlamp in a given spectral band, making it highly suitable for many specific skin treatments.

**Fig 12:**
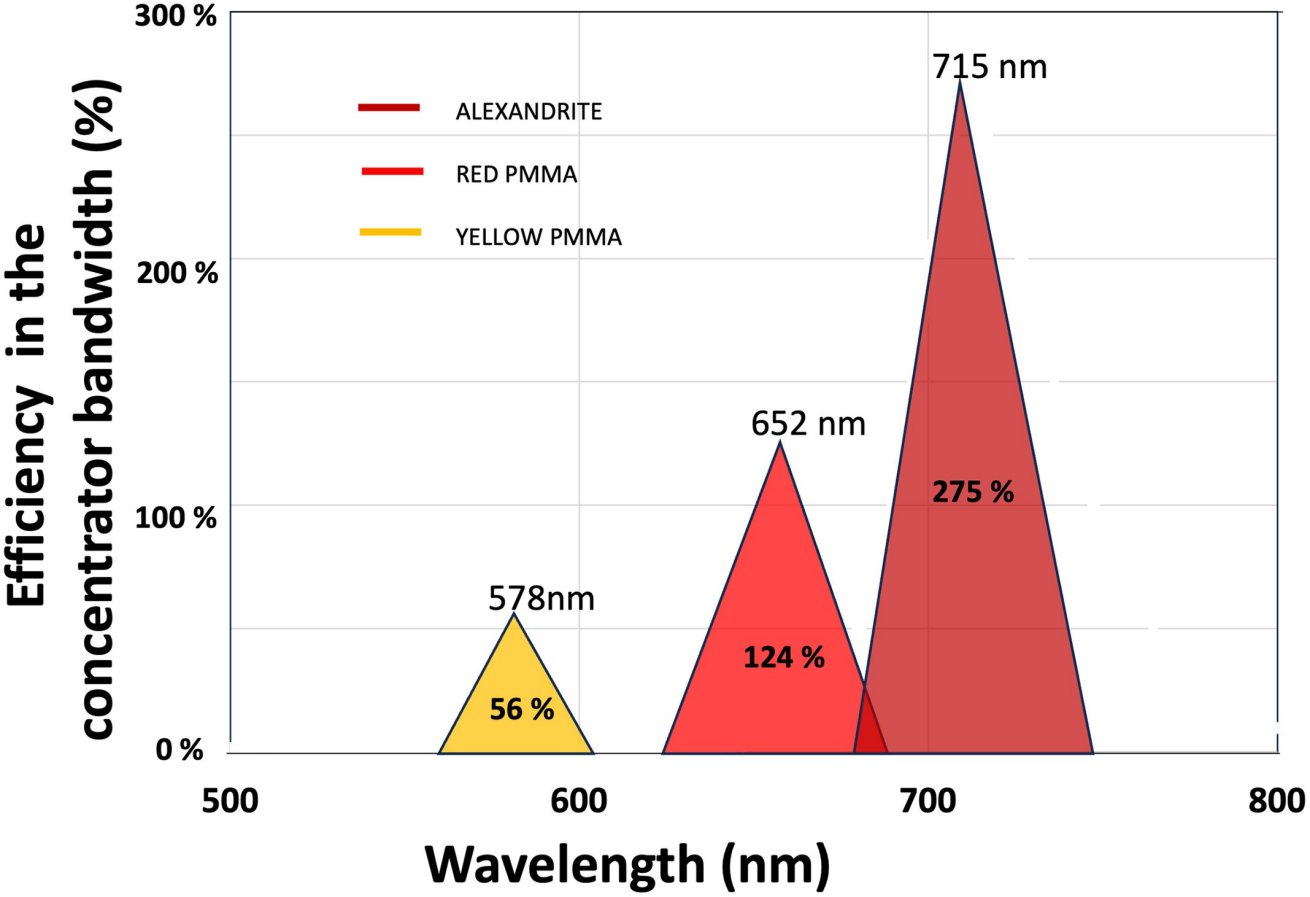
Illustration of Table 1. Comparative analysis between the IPL flashlamp and our various FLCs, specifically focusing on efficiency within the concentrator bandwidth.

To provide a more accurate assessment of the spectral concentration of the FLCs, we calculate the power of the IPL within the spectral band of each FLC, as defined by its full width at half maximum. We then define the efficiency within the FLC bandwidth as the ratio of the power emitted by the FLC to the power of the IPL within the FLC spectral band. These values are presented in Table 1 and illustrated in Fig 12. Within the specific band of each FLC, the efficiency is 56% for the yellow FLC, reaching 124% for the red FLC and even 275% for the alexandrite FLC.

**Table 1:** Comparative analysis between the IPL flashlamp and our various FLCs. The IPL Fluence is calculated in each FLC bandwidth.

	FWHM emission bandwidth	Central wavelength	Output Fluence	Calculated IPL fluence in the FLC emission bandwidth	Efficiency in the concentrator’s bandwidth
**IPL XENON FLASH**	168 nm	520 nm	18.5 J/cm^2^		
**YELLOW FLC**	30 nm	578 nm	2.3 J/cm^2^	4 J/cm^2^	56%
**RED FLC**	50 nm	656 nm	3.3 J/cm^2^	2.6 J/cm^2^	124%
**ALEXANDRITE FLC**	54 nm	717 nm	4.5 J/cm^2^	1.6 J/cm^2^	275%

The first column of Table 1 denotes the full width at half maximum (FWHM) emission bandwidth, while the second column indicates the central wavelength for each FLC. The third column provides the output fluence of the FLCs, measured for a given pump fluence of 18.5 J/cm^2^. According to Fig 12, the three measured fluences at the output of the FLCs are reported here. The fourth column displays the calculated IPL fluence within the emission bandwidth of each concentrator. This last column demonstrates that the fluence significantly increases when reaching the red bandwidth and even reaches 275% efficiency for our alexandrite FLC.

The second series of measurements builds upon our results obtained with the Nordlys^®^ from Candela^™^ system using the PL400 handpiece. For these experiments, we relocated our three FLC kits to the St. Etienne CHU hospital in collaboration with the dermatologist Professor Jean-Luc Perrot. This on-site approach enabled us to evaluate the performance in a real clinical setting.

Our experimental procedure involved attaching the FLC to the PL400 handpiece. This handpiece is well suited for pumping our three luminescent concentrators as its spectrum bandwidth corresponds to the xenon flashlamp bandwidth in the range of 420 nm to 720 nm. We managed to vary the IPL fluence from 2.5 J/cm^2^ to 7 J/cm^2^ with a 2.5 ms pulse duration. This allowed us to utilize our Gentec joulemeter to measure the energy at the output of the light guide.

**Fig 13:**
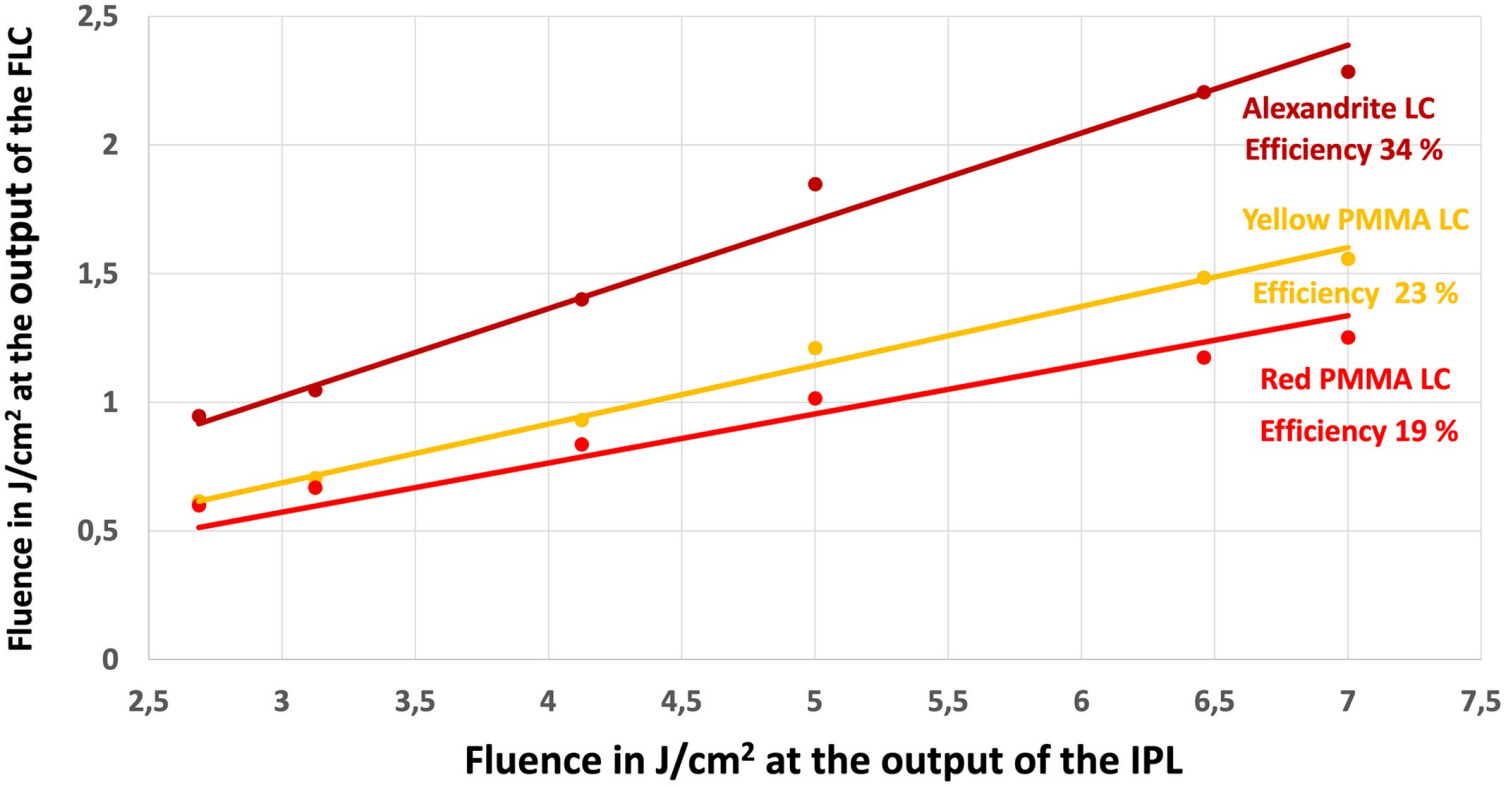
Output fluence of FLCs with Nordlys^®^. Experimental results were obtained for the three FLC colors (alexandrite, red and yellow PMMA Concentrators) using the Nordlys^®^ from Candela^™^ system with the PL400 handpiece (48 mm by 10 mm). The efficiency varied from 19% to 34% for the alexandrite and is much higher than using the MED-230 IPL.

The efficiency achieved for each FLC is even greater than what we observed with the MED 230 IPL system (Fig 13). The result with the alexandrite shows that more than a third of the fluence delivered by the IPL is transported to the output of our fiber. This improvement is attributed to a more favorable matching between the spectral emission of the flashlamp and the spectral absorption characteristics of each concentrator.

### Discussion and comparison with IPL and lasers

The fibered luminescent concentrator has limitations that must be considered in order to identify potential applications.

The first limitation is the energy available at the output of the fiber: compared to an IPL, the output energy is almost two orders of magnitude lower. If we take into account the reduction factor of the surface area with our FLC (5 mm diameter, surface area of 0.2 cm^2^) compared to a typical handpiece (surface area of 5 cm^2^), the fluence is typically only 3 to 5 times lower, as shown in Fig 10. The fluence is what usually matters in terms of treatment efficiency. In our study, the maximum fluence of 6 J/cm^2^ was obtained with our FLC at a pulse duration of 9 ms. This optimum fluence can be increased by broadening the pulse train to a higher value. Unfortunately, our IPL wasn’t programmed to deliver longer pulses. An example of using longer pulses has been demonstrated for the treatment of nasal telangiectasias by E. Geddes-Bruce et al. in reference [14]. They have demonstrated that due to the large thermal relaxation time of large blood vessels like nasal telangiectasias, it is better to deliver longer pulse durations of up to 40 ms. For a pulse duration of 40 ms, the fluence achievable by our system can reach a value of more than 25 J/cm^2^, which corresponds to the treatment range.

Indeed, FLCs can achieve higher fluence in a specific spectral bandwidth compared to IPL and with a more precise spectrum. This implies that illumination doses with FLC can be more efficient than those of IPL, leading to a significant reduction of unnecessary photons that may induce heat and pain for the patient. As an example, we can mention the paper by R. Wanitphakdeedecha [5] on the efficacy of using a 577 nm laser for post-acne erythema. They used a 1 mm spot size, 12–15 J/cm^2^, 30 ms laser to target oxyhemoglobin and demonstrated that the yellow laser is ideal for treating vascular lesions. Our FLC apparatus, utilizing a yellow PMMA, could be perfectly adapted for such treatment and is certainly less expensive. FLC possesses the versatility to cover a wide range of wavelengths, ranging from yellow to dark red. In addition, we demonstrate that it can be adapted for different types of IPL, simply by swapping the FLC kit on the IPL handpiece.

FLCs are not well-suited for treating large surfaces like IPL. They are primarily designed for applications that demand precision. The benefits include a smaller spot size and a significantly lighter handpiece, providing the same level of convenience as lasers. The small energy on a divergent output beam makes FLC much safer than IPL and lasers, which is a crucial safety consideration in medical and cosmetic procedures.

Another limitation comes from thermal effects. In the case of PMMA, the repetition rate at maximum energy cannot be sustained at 2 Hz for more than 10 seconds due to heating issues. However, the lifetime at 1 J/cm^2^ (corresponding to a pump fluence of 5 J/cm^2^) is estimated to be much higher. We haven’t noticed any degradation after 1000 of shots at 1Hz.

Nevertheless, it is important to highlight that replacing the plastic material is a straightforward and cost-effective solution in cases where excessive heating causes performance degradation. In addition, these effects can be managed by improving the cooling of the PMMA or by replacing plastics with materials having higher thermal conductivity, such as crystals. In this regard, the alexandrite FLC exhibits much better thermal behavior, showing no signs of degradation.

FLC serves as an additional component for an IPL device, positioning itself as an intermediary between lasers and IPL. Importantly, it does not replace either system or technique but rather augments them, functioning as a specialized tool. The choice of luminescent concentrator determines the emission wavelength and can be tailored to the specific therapy or treatment requirements. The FLC’s design is straightforward, comprising luminescent concentrators powered by an IPL laser head and connected to a light guide for precise skin interaction.

To summarize, we provide a comparative table (Table 2) of the technical advantages and disadvantages of the two most commonly used techniques in light therapy: the laser and the IPL.

These points highlight the importance of the FLC innovation, which combines the benefits of both techniques.

**Table 2:** Comparison of the technical performance of lasers, IPL, and FLC for potential use in light therapy.

DEVICE	LASER	IPL	FLC
**Simple technique**	-	+	+
**Low cost**	-	+	+
**Spectral performance**	+	-	+
**Temporal performance**	+	+	+
**Ease of handling, lightweight**	+	-	+
**Spectral availability for each treatment**	-	-	+
**Action on a small surface**	+	-	+
**High beam divergence, low danger**	-	+	+

The spectral performance of an FLC is comparable to that of a laser, allowing for targeted action similar to a laser, which is not typically the case for a traditional IPL. The use of a fiber to transport light, as in a laser, offers increased maneuverability of the handpiece, thereby eliminating the need for water cooling and high-voltage transport to the flash, as required by IPL handpieces.

The FLC technique is simple to implement as it adapts to any IPL handpiece. The temporal performance (square pulses, long pulses) is identical and without deformation as with an IPL.

The integration of a luminescent concentrator with an associated collimator and a liquid light guide allows for the transport of a large amount of energy, thus approaching the energy performance of IPLs.

The FLC technique is not a laser and therefore does not have the same dangers. The beam exiting the fiber is extremely divergent, like any incoherent light, similar to that of an IPL.

## Conclusion

In summary, we have demonstrated a novel light source based on a flashlamp-pumped luminescent concentrator design. The fibered luminescent concentrator utilizes light guides to deliver a small spot (5 mm diameter) of controlled spectrum (30–50 nm FWHM) with performance that can surpass that of an IPL, with a significantly higher level of safety. The selection of a luminescent concentrator determines the emission wavelength and can be customized to meet the specific therapy or treatment requirements. In this paper, we demonstrate for the first time that doped PMMA can be utilized for luminescent concentration under flashlamp pumping in the yellow and red wavelengths. It is also the first time that an alexandrite crystal, renowned for its laser performance, has been employed for spontaneous emission in the "dark red" spectrum (690–740 nm) as a luminescent concentrator. In all cases, the performance reaches the level of J/cm^2^ for 9 ms pump pulses, which is sufficient for skin treatments.

The design of the FLC is simple, consisting of a luminescent concentrator powered by an IPL laser head and connected to a light guide for precise skin interaction. It is highly versatile and safe in terms of emission wavelength and pump source. The FLC could serve as an additional component to an IPL device, positioning itself as an intermediary between lasers and IPL. Importantly, it does not replace either system or technique, but rather complements them, acting as a specialized tool.

Fibered Luminescent Concentrator could indeed have a bright future.

## Supporting information

S1 Data (CSV)

S2 Data (CSV)

S3 Data (CSV)

S4 Data (CSV)

S5 Data (CSV)

S6 Data (CSV)

S7 Data (CSV)

S8 Data (CSV)

S9 Data (CSV)

S1 Table (DOCX)
