# Supplementary material for: Fibered luminescent concentrator: A bridge between flashlamp devices and laser technologies for skin therapy
Source: PLoS One. 2024 Dec 18;19(12):e0311425. doi: 10.1371/journal.pone.0311425 (PMC11654970; doi:10.1371/journal.pone.0311425)
Supplement: Supplementary file 1 [file pone.0311425.s010.docx]

	FWHM emission bandwidth	Central wavelength	Output Fluence	Calculated IPL fluence in the FLC emission bandwidth	Efficiency in t[illegible] concentrator[illegible] bandwidth
**IPL XENON FLASH**	168 nm	520 nm	18.5 J/cm^2		
**YELLOW FLC**	30 nm	578 nm	2.3 J/cm^2	4 J/cm^2	56 %
**RED FLC**	50 nm	656 nm	3.3 J/cm^2	2.6 J/cm^2	124 %
**ALEXANDRITE FLC**	54 nm	717 nm	4.5 J/cm^2	1.6 J/cm^2	275 %
